# Gap Analysis of Ambient Electromagnetic Noise Measurements Stored in the ITU Data Banks

**DOI:** 10.3390/s24216832

**Published:** 2024-10-24

**Authors:** Ben A. Witvliet

**Affiliations:** 1 Radio Systems, Faculty of EEMCS, University of Twente, P.O. Box 217, 7500AE Enschede, The Netherlands; b.a.witvliet@utwente.nl; Tel.: +31-6-1219-0688; 2 Dutch Authority for Digital Infrastructure, Ministry of Economic Affairs, P.O. Box 450, 9700AL Groningen, The Netherlands

**Keywords:** ambient, electromagnetic noise, radio noise, man-made noise, EMC, measurement, ITU, Study Group 3, Recommendation ITU-R P.372, Recommendation ITU-R SM.1753

## Abstract

For any radio frequency (RF) sensor (receiver) to function optimally, the ambient noise field strength, converted to electrical power by the transducer (antenna), must be lower than the in-ternal noise of that sensor. Therefore, knowledge of the expected ambient noise level is essential for the design of sensors for earth observation, atmospheric research, radio astronomy or navigation. The International Telecommunication Union (ITU) provides a model that predicts ambient man-made noise levels, differentiated by frequency, origin and environment. This is entirely empirical model is based on data from the 1960′s and 1970′s. In recent years, 90,205 noise measurements have been collected to update the model. The analysis of that data set presented here is essential as it shows a pitfall to avoid: despite to size of the data set it is sparce over the parameter space, and unacceptable biases occur when a purely empirical model is based on them. The paper proposes another approach: to create a mathematical model based on physics that can be fine-tuned and validated using these collected measurements, without producing the biases. A revolutionary side effect of such a model would be the linking of two currently isolated domains, that of spectrum management and electromagnetic compatibility.

## 1. Introduction

Ambient electromagnetic noise or ‘radio noise’ is the main limiting factor for advanced sensor systems that observe weak signals below 20 MHz, even in quiet rural locations. At these frequencies, at least when using an efficient transducer (antenna) of the sensor, the power produced by the external noise field exceeds that of the internal noise generated by the first amplification stages [1] (p. 766). In densely populated areas, the dominance of radio noise may extend beyond 500 MHz [2]. Examples of such sensor systems are radio equipment for security, health and safety, navigation [3] radio interception and direction finding [4]. Scientific instruments for radio astronomy [5], and for upper atmospheric and space weather observations—such as ionosondes, RIO-meters, polarimetric sensors [6] and incoherent scatter radars—may also be affected.

### 1.1. Natural Sources of Radio Noise

In quiet rural areas, where the population is sparse, most of the radio noise received originates from remote sources. For example, the noise from distant lightning discharges transported via the ionosphere [7], emissions from extraterrestrial sources such as Jupiter [8,9], the Milky Way [10] and the Sun [9,11]. The noise from lightning discharges [12] is impulsive in nature, although the impulses may have spread in time due to dispersion in the ionosphere when they arrive at the sensor. The emissions from Jupiter, the Milky Way and the Sun appear as band-limited Gaussian noise, with a wideband but distinct frequency profile. Additionally, Jupiter shows random emissions at frequencies between 20 and 40 MHz, generated in its magnetosphere, which sounds like a hiss, with incidental popping modulations.

### 1.2. Man-Made Radio Noise

The radio noise environment is very different in cities and residential areas, where the population is dense, and where the density of electrical and electronic devices is high. Each of these devices emits a small amount of electro-magnetic radiation, and all these components add up to an electromagnetic field that resembles additive white Gaussian noise (AWGN) [13]. The cumulative field strength of this ‘man-made noise’ is significantly higher than the natural noise. Often an impulse noise (IN) component maybe superimposed on the AWGN component, the amplitude of which will depend on the pulse width of the source and the sensor (receiver) bandwidth. If there is one or a small number of man-made noise sources nearby, single-carrier noise (SCN) may be seen in the spectrum, consisting of one or multiple narrow-band noise-like signals, carriers or noisy spectral ‘humps’ [14]. Especially in an indoor environment the likelihood of one or a few sources being in close range is high, and as a result a mix of AWGN, IN and SCN is observed.

### 1.3. Radio Noise Level Predictions

When the ambient noise is indeed higher than the internal noise generated by the sensor, knowledge of the expected radio noise level is important for system design, as it factors in the link budget calculations. Additionally, when compatibility studies are performed for the assignment of frequency spectrum to new radio applications, the ambient noise level is often taken as a reference level. Unwanted emissions at levels below the expected radio noise level are then considered to be no nuisance to the incumbents. For these reasons, the Radio Sector of the International Telecommunication Union (ITU), the organization of the United Nations responsible for world-wide radio spectrum management, maintains Recommendation ITU-R P.372 [15]. This “recommendation”—it is actually more of a standard rather than a recommendation—contains information on the expected levels of radio noise generated by different natural and man-made sources. Of course, due to its wide spectral and geographic coverage (all frequencies, the entire world), the information in this Recommendation cannot be overly specific and precise. Still, it is considered the authoritative reference on radio noise world-wide. The current version of the Recommendation is version 17. In this article, it will be referred to as ‘P.372’.

### 1.4. The Desire to Update P.372 

The predicted man-made noise levels in P.372 were derived from a limited set of measurements performed between 1950 and 1970 and has since remained unaltered. However, since that time, the number of electrical and electronic appliances has increased exponentially, and one would expect the man-made noise to increase with the number of appliances. On the other hand, the introduction of EMC (electromagnetic compatibility) requirements could have mitigated this increase. Objective information to this end is scarce.

From the side of equipment manufacturers, there is a constant pressure to increase the predicted man-made noise levels in P.372. Their reasoning is that higher predicted levels of man-made noise in P.372 will allow higher levels of unwanted emissions in compatibility studies and higher emissions levels in EMC norms, as these emissions will be masked by the already existing man-made noise levels. And allowing higher unwanted emissions will make the design and production of their products cheaper. However, the P.372 curves are no protection curves, but they describe a current, potentially unwanted, situation. Some spectrum users—the victims of increased noise levels—also promote increasing the predicted man-made noise levels in P.372. They hope that raising the noise levels in P.372 will alert the authorities and result in stricter EMC and unwanted emission regimes to reduce the man-made noise. The latter is a misconception: the given noise levels are taken as a reference in compatibility studies without further discussion about trends and the necessity to act on them.

### 1.5. The Current Model Is Empirical

The man-made noise values given in P.372, on frequencies up to 300 MHz, are based on measurements data dating back half a century. P.372 provides an empirical man-made noise model. Only one model is available, supposedly valid for the entire world. This does not seem very realistic, as equipment emissions will be higher in countries with less stringent emission rules or enforcement of these rules. And regional differences in construction style and material will influence the wall attenuation of buildings [16], and hence the emission per house. It would therefore seem logical to include parameters in these models based on the local situation. So far, this is not the case. In the last two decades, several member states have provided man-made noise measurements to the ITU Radio Noise Data Bank, and a few attempts have been made to fit curves through these data points to update the model in P.372. So far, these attempts have not been successful. In fact, it can be shown that corruption of the man-made noise model is very likely, unless the distribution of the measurement data over measurement year, frequency spectrum, geographical location, and environment category is accounted for. 

This article opposes this approach. It will be shown that using the data indiscriminately to construct an empirical model will result in strong biases as a result of the uneven distribution of the measurements over the globe, and due to data scarcity in several dimensions of the data set. A better approach to arrive at a trustworthy man-made noise model will be proposed, along with other suggestions to improve the quality of the ITU radio noise data set.

The article is structured as follows. Section 2 introduces the ITU radio noise model, the desire to update this model and the measurement data collected in the ITU Radio Noise Data Bank. This data set is analyzed for gaps in geographical coverage, coverage of environment types, spectral coverage and temporal distribution in Section 3. As discussed in Section 4, it will be shown that despite its size, this data set must be considered too sparse for the creation of an empirical model, and a better approach is proposed in Section 5. Conclusions can be found in Section 6.

## 2. Man-Made Noise Levels in P.372

**Figure 1:**
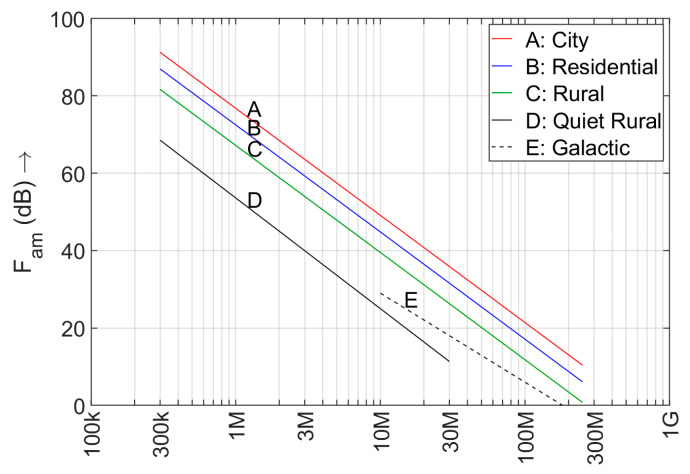
Man-made noise levels as a function of frequency for 4 different environments, according to P.372-17 [15]. The median value of the AWGN component is shown. The Galactic noise level shown will be dominant at frequencies where the ionosphere is (partly) transparent.

In P.372, the radio noise level is expressed as an ambient noise factor *Fa*, which is the ratio of the power output *P_n_* of a lossless reference antenna and the thermal noise power *kTB*. In logarithmic form, expressed in decibels:

*F_a_* = *P_n_* − 10 × *log*_10_ (*k T B*) (1)

where *k* is Boltzmann’s constant (1.38 ×10^-23^ J/K), *T* is the temperature (assumed 290 K), and *B* is the bandwidth in Hz. The reference antenna is a ground-mounted short lossless vertical monopole, or a lossless resonant half-wave dipole antenna. The predicted median noise factors for man-made noise are given in Figure 1. They can also be found in the form of a formula and a table in Part 6 of the Recommendation. The value shown represents the relative power of the background radio noise, which is assumed to resemble AWGN. The power spectral density of this component is independent of the sensor bandwidth. Radio noise is measured with a Root Mean Square (RMS) detector, preferably in a bandwidth that allows separation of the background noise from radio signals [13]. Intentionally emitted radio signals are not noise and should not be included in the measurements. Four environmental categories are shown in Figure 1: City, Residential, Rural and Quiet Rural. According to P.372, the difference between City and Quiet Rural is 24 dB, the difference between Rural and Quiet Rural is 14 dB. A future revision of the standard will probably use new environment definitions proposed in Recommendation ITU-R SM.1753 [17], in which Remote Rural replaces Quiet Rural and a few new categories are added. The given values are for outdoor man-made noise. 

Especially the Quiet Rural category is important for spectrum management: this is the level of radio noise when few or no man-made noise sources are nearby. This may be considered the base-line radio noise level.

## 3. Gap Analysis

To see whether the existing model can be updated using new empirical data, the ITU data set is analyzed for gaps in its coverage. The raw data used in this paper is obtained from the Radio Noise Data Bank of ITU-R Study Group 3. This analysis is restricted to the outdoor man-made noise measurements contained in the database. Currently, as of 1 October 2024, the ITU Radio Noise Data Bank contains a total of 90,205 such measurements. Each measurement is characterized by its observation frequency, country, location, environment type, measurement year, and time-of day. Although the total number of measurements is large, it will be shown that the data set is scarce in this multi-dimensional space. In this section we will analyze the dataset. The impact of data gaps will be dicussed in Section 4. 

### 3.1. Geographical Distribution of Measurements

**Figure 2:**
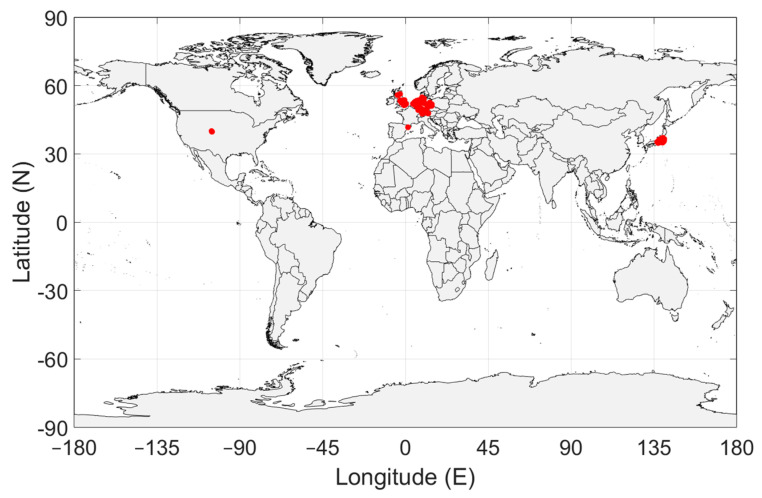
Geographical distribution of the man-made noise measurements.

The geographical distribution of the measurements is shown in Figure 2. This map shows the first major deficiency of this data set. Only measurements in Europe, Japan and the USA are available. These are all rich and highly industrialized countries, data from Lower and Middel Income Countries (LMIC) is missing. Approximately 80% of the measurements have been done in Japan, see Table 1. The impact of these observations will be discussed in Section 4.

**Figure 3:**
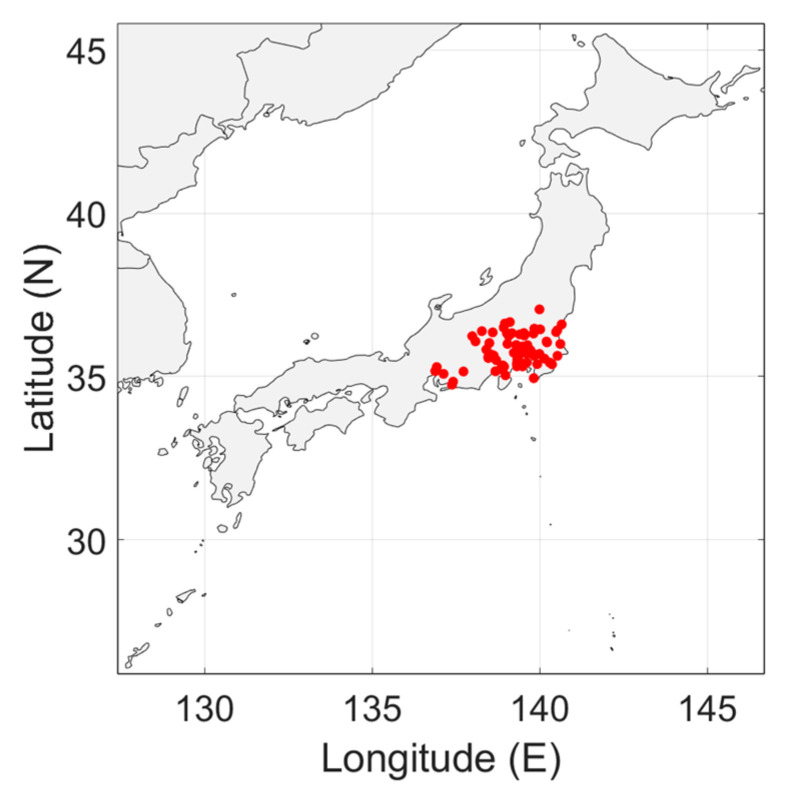
Geographical distribution of the man-made noise measurements in Japan. The measurements cover the central part of the Honshou island.

**Figure 4:**
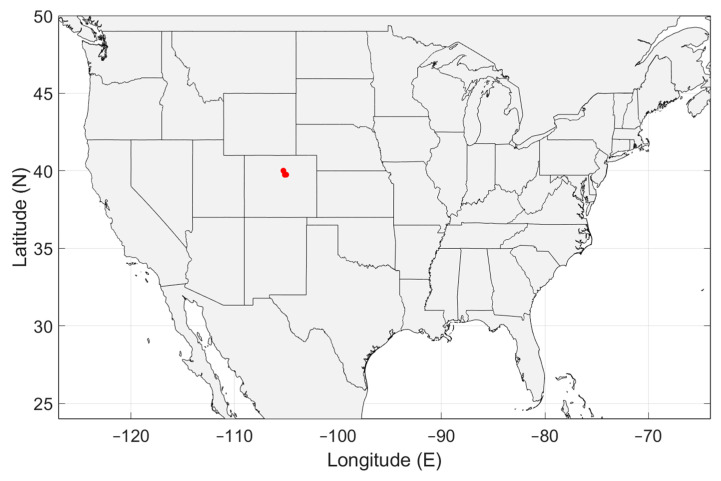
Geographical distribution of man-made noise measurements in the United States of America (USA). The measurements were done on two locations near Boulder, CO, USA.

**Figure 5:**
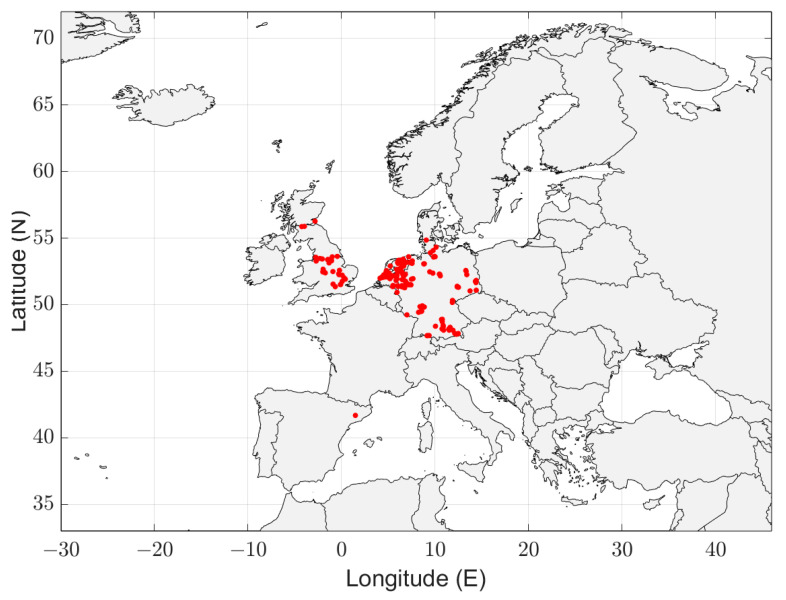
An overview of the geographical distribution of the man-made noise measurements in Europe. The measurements were done in the United Kingdom, Germany, The Netherlands and Spain.

**Figure 6:**
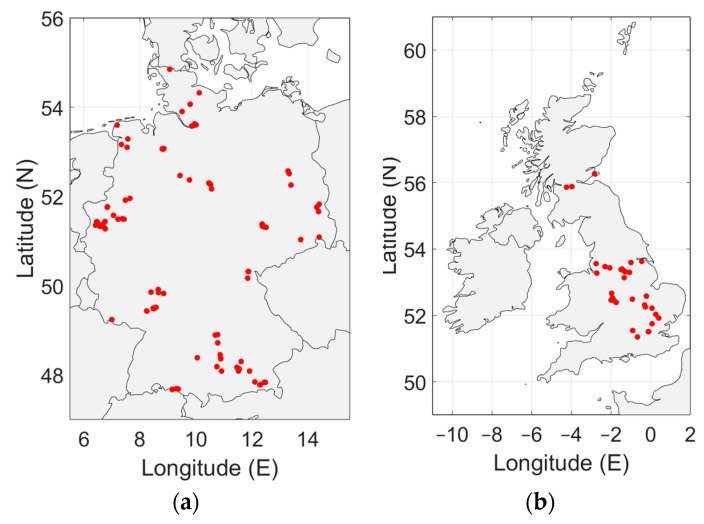
(**a**) Geographical distribution of the man-made noise measurements in Germany. No measurements were made in Quiet Rural areas, as this exceeded the capability of the equipment. (**b**) Geographical distribution of the man-made noise measurements in the United Kingdom.

**Figure 7:**
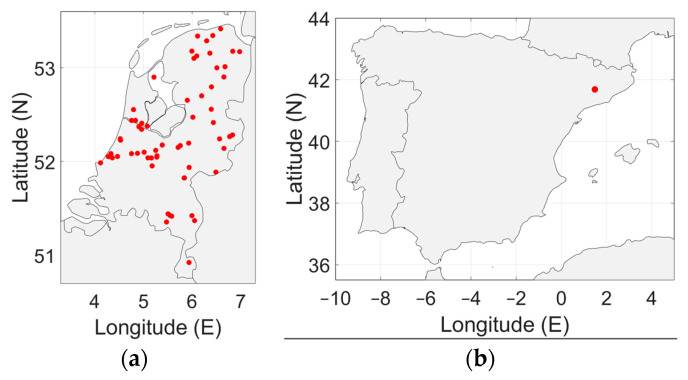
(**a**) Geographical distribution of the man-made noise measurements in the Netherlands. (**b**) Geographical distribution of the man-made noise measurements in Spain (single location).

To accurately represent the radio noise level in a country, the measurements must be distributed evenly over the country. In Figure 3, Figure 4, Figure 5, Figure 6 and Figure 7, the geographical distribution of the measurements in each country is shown.

In Europe, the measurement locations do not evenly cover the continent. South Europe, Eastern Europe and Scandinavia are not represented. It can be seen in Figure 4 and Figure 7b that the geographical spread of the measurement locations is especially poor in Spain and in the USA. The number of measurement locations in each country is given in Table 2. Approximately 79% of the measurement locations are in Germany, see Table 1. The impact of these observations will be discussed in Section 4.

### 3.2. Measurements per Environment Type

As the current empirical man-made noise model in P.372 differentiates between 4 environment types, the individual measurements in the Radio Noise Data Bank also contain labels describing the local environment type. Table 3 and Table 4 provide an overview of the absolute and relative number of measurements per environment type per country. 

In the Radio Noise Data Bank, the environment types are labeled according to the definitions given in Recommendation ITU-R SM.1753:
REMRRemote Rural (P.372: Quiet Rural)RURLRuralRESDResidentialCITYCityURBNUrbanINDAIndustrial AreaROADHighwayOFFCOffice area

From Table 4 it becomes clear that not every country equally distributed their measurements over locations in the different environments. The dominant contribution for each environment is highlighted. 

### 3.3. Spectral Coverage of the Measurements

**Figure 8:**
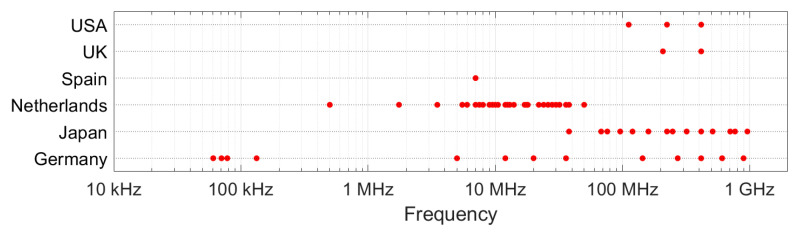
Spectral distribution of the man-made noise measurements per country. Red dots show the frequencies at which measurement data are in the Radio Noise Data Bank.

The man-made noise level is frequency dependent. A close look at the data set reveals that the number of frequencies observed is limited and that not every country has measured over the entire spectral range. The USA, UK and Spain measured on 3 frequencies or less. This is shown in Figure 8. Table 5 shows the number of frequencies per environment in each of the countries. 

### 3.4. Temporal Distribution of Measurements

**Figure 9:**
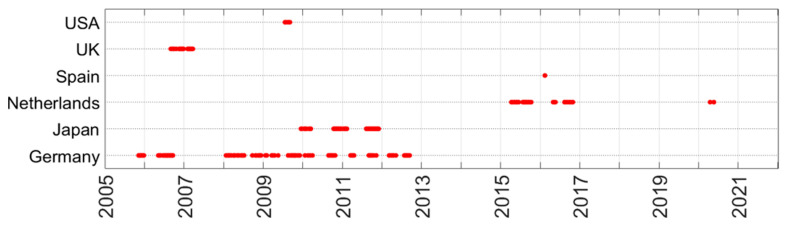
Temporal distribution of the man-made noise measurements per country.

Figure 9 shows the distributions of the measurements over time. Consistently repeated measurements were only done in Germany between 2006 and 2013. 

## 4. Discussion and Recommendation

In this section, the impact will be discussed of the gaps in the coverage of the parameter space that was observed in previous section. The principle questions asked are: (1) If the new ITU man-made noise model would be entirely empirical, what will be the impact of the data gaps? (2) And considering these gaps, what alternative approach may be recommended?

### 4.1. Impact of Insufficient Geographical Coverage

Table 1 and Figure 2 show that all measurements are in rich and highly industrialized countries, and none is in Lower and Middel Income Countries (LMIC). Therefore, if these data would be used as sole input to derive an empirical radio noise model, a strong bias towards rich, industrialized countries may be expected. This would no be a problem if the man-made noise situation in these countries could be thought representative for the entire world. However, several important parameters that influence the local man-made noise level may differ significantly between these countries and e.g., LMIC. Especially the difference in equipment quality, the electromagnetic compatibility (EMC) regime and its enforcement, the electromagnetic shielding of buildings (wall attenuation) [16] and the unwanted transmission via electricity wiring. But also when regarding other countries, the influence of people density and distribution, equipment density per dwelling is not expected to be negligible.

Table 1 reveals that 80% of the measurements is done in Japan. Therefore, if all measurements would be processed without differentiating between the data sets of the individual countries, the Japanese radio noise environment would have a dominant influence on the empirical model. In fact—if we ignored the limited spectral coverage, which will be discussed later—the model would predict the radio noise environment in Japan, not that of the rest of the world. Of course, this is not the goal of the model.

As Figure 3, Figure 4, Figure 5, Figure 6 and Figure 7 show, the distribution of measurement locations each the countries also differs in quality. The geographical spread of the measurement locations is especially poor in Spain and in the USA. If we look at the number of measurement locations regardless of the country, Germany is overrepresented (79%), see Table 1. Therefore, if we would not take individual measurements but measurement locations as a basis—e.g., because of measurements at multiple frequencies in each location—the data of Germany would become dominant. 

### 4.2. Insufficient Coverage of Environment Types

Above, we assumed that all measurements (or all measurement locations) could be used to form an empirical model. However, if we have to create separate models for each environment (see Figure 1 and Section 3.2), the data set will be split over the different environments. From Table 4 it becomes clear that not every country equally distributed their measurements over locations in the different environments. 

Therefore, when subsequently the empirical data would be used to generate a man-made noise model for each environment type, each model would have a strong bias towards a single country. The remote rural model would represent the Netherlands, the office area model would represent the UK, and the other models would represent Japan. 

The uneven spread of the measurements over different environments is explainable. The preference for measurements in certain environments is driven by the practical need for man-made noise data in these countries. Also, it is not easy to design a system that is sensitive enough to accurately measure Quiet Rural noise levels, as the system must be highly linear to withstand the very strong radio signals in the same or adjacent frequency bands. The German measurement system, for example, did not have the ability to measure Quiet Rural levels. 

Of course, if the noise levels would be the same in these environments regardless of the country, the result would be consistent and usable. But this is not very likely, ans such an assumption would have to be proven first. Our expectation is that differences between these 6 countries, and even more so the significant differences with LMIC’s, may require an extra parameter (or several) to account for differences between individual countries and regions. However, despite the number of measurements, the data set is too scarce to determine these parameters empirically.

### 4.3. Insufficient Spectral Coverage

The unwanted emissions of electronic and electric equipment is generally stronger on lower frequencies and the propagation of man-made noise from the noise source to the sensor depends on the propagation, which is also frequency dependent. Consequently, the man-made noise level will be frequency dependent. There is some debate however, whether the straight parallel lines in the double-logarithmic graph of Figure 1 correctly represent the debit of the noise level with increasing frequency. 

When regarding the high number of measurements (90,205), one would expect sufficient measurement points to redraw these graphs. However, a closer look at the data set reveals that the number of frequencies observed is limited and that not every country has measured over the entire spectral range. This is depicted in Figure 8. Table 5 shows the number of frequencies per environment in each of the countries. The USA, UK and Spain measured on 3 frequencies or less. This spectral data scarcity makes it very difficult (or impossible) to derive good models from the empirical data alone.

It is noteworthy that one major shortcoming of the data sets is caused by the tendency to measure very few frequencies in a frequency range of interest. This is often done because of efficiency or due to technical limitations. This makes it impossible to capture the relationship between noise level and frequency. If time is limited, instead of doing 10,000 measurements always on the same 3 frequencies, it would be much more productive to select 3 different random frequencies at each measurement location, as that will provide a picture of the overall spectrum of the radio noise.

### 4.4. Impact of Insufficient Temporal Coverage

As briefly mentioned in the Introduction, both spectrum users and regulators would be interested in trend information, preferably in de form of a graph showing the increase or decrease of the radio noise over time. This information could be used to either tighten or relax the regulations on unwanted emissions. To provide that information, consistent and repeated measurements over time are needed. Figure 9 shows the distributions of the measurements over time. Unfortunately, consistently repeated measurements were only done in Germany between 2006 and 2013. They will allow (local) trend analysis over that interval only and only in one country.

### 4.5. A Recommendation for Improved Participation of External Organizations

The measurements stored in the Radio Noise Data Bank are certainly not the only radio noise measurements done world-wide. Unfortunately, data of several very interesting and valuable radio noise studies have never been submitted to the ITU Radio Noise Data Bank. Outside the ITU, the existence of this data bank is not widely known, and currently it is not very easy to access from outside the ITU, which will hopefully be improved soon. However, Study Group 3, the group that creates and manages the propagation models and the radio noise models of the Radio Sector of the ITU (ITU-R), welcomes such input. Of course, any submission must fulfill high quality standards, to avoid pollution of the data bank with flawed data, and adopt the required data format. Measurement system requirements and data formatting is described in Recommendation ITU-R SM.1753 [17], but it must be said that this manual needs updating, so that it will focus more on how to establish and report the performance and measurement uncertainty of the system, instead of describing standard system components, so that it will become more future proof and more inclusive of innovative and state-of-the-art solutions. Currently, the Radio Noise Data Bank is managed by Ofcom, UK, who can provide more information on required data quality and data submission. Further information can always be obtained by contacting the chairpersons of Study Group 3 [18].

### 4.6. A Recommended Alternative Approach to Establish an ITU Noise Model

Considering the insufficient coverage of this (large) data set over the entire parameters space, one must conclude that it is impossible to generate a purely empirical model from this data. However, even though we do not know the difference between each country that we discussed, we can assign parameters to them. And if we understand the physical nature of the propagation and accumulation of the noise from a large number of noise sources, we can make a physical model of the man-made noise. When this model sufficiently approaches reality, the collected measurements in the RNDB can be used to validate the model. This approach will be described in the next section.

## 5. Future Work: A Physical Model, Validated with Measurements

The creation and validation of the physical model is part of ongoing research that will be published in a separate paper. However, the design principles will be detailed here to encourage others to look along similar lines and improve on it. 

**Figure 10:**
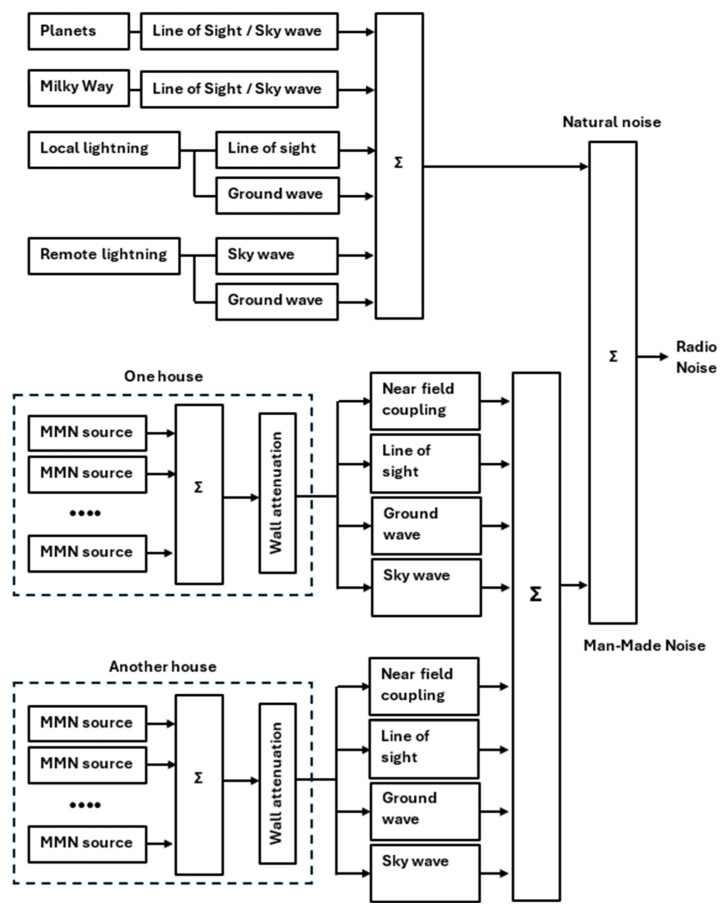
A physical model of radio noise arriving at the sensor.

The physical model could may its input from the building density, the density of devices per building, the emitted effective isotropic radiated power (EIRP) of each device, the distance from the buildings to the sensor, the propagation paths on the frequency of observation. There will be multiple propagation paths from source to sensor possible: direct coupling in the Near Field, Line-of-Sight, ground wave, sky wave. These propagation paths were described in [13,14] and can be seen as parallel paths to the sensor, see Figure 10. This model will be supported mathematically, using existing models for radio wave propagation and wall attenuation. 

**Figure 11:**
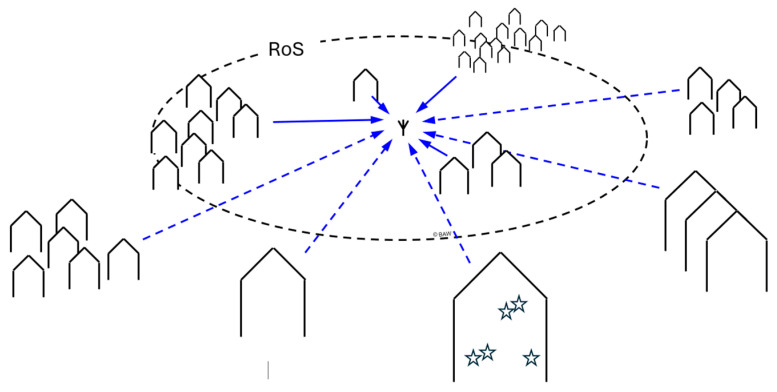
The Radius of Significance (RoS) contains the sources that predominantly define the man-made noise level at the sensor. The outlined shapes represent houses, the stars in one house depicture that each house contains multiple sources.

Which path is dominant for each source will depend on distance and frequency. To obtain the noise level, the number of buildings within the Radius of Significance (RoS), the radius around the sensor beyond which the contribution of more noise sources will become negligible, must be known. This is depicted in Figure 11. The number of buildings may be an educated guess, or it can be obtained using a Geographical Information System (GIS). The dominant propagation mechanism determines the RoS, and therefore this distance is frequency dependent. While not named as such, a first hint towards the concept of the RoS can already be found in the thesis of Fockens [19], who showed a correlation between the number of houses in a circle of a certain radius and the measured man-made noise level. Breton, Haedrich, Kamrath and Wilson, on the other hand, modelled the distribution of urban man-made noise levels across an area [20].

To evaluate model performance, a large number of measurement location coordinates may be randomly selected from the Radio Noise Data Bank (RNDB) and compared with model output for the same location. The root-mean-square error (RMSE) of the difference may serve as a figure-of-merit. Further test on random points of selective subsets of the RNDB data may be necessary, to prevent that a low overall RMSE is achieved, while performance is poor in in specific regions of the parameters space.

## 6. Conclusions 

### 6.1. RNDB Data Set Should Not Be Used for an Empirical Model

As was shown, blindly combining measurements and ignoring that these data sets were obtained in different countries, in different environments, on dissimilar frequencies, and in different years, is not a good idea. This will introduce a strong bias towards certain countries for subsets of the model, and result in an inconsistent model that does not represent reality. Furthermore, it was noted that all measurements come from rich and industrialized countries. Data from large parts of the globe, especially from LMIC countries, are missing. This makes it impossible to create an empirical new model that represents all countries based on these measurements. 

### 6.2. Application of the Model to Regulatory Discussions

With a physical model as described in previous section, a direct relationship between the ambient electromagnetic noise level and the unwanted emissions of electrical and electronic equipment will be established. Therefore, the creation of such a model and validating it with (existing and new) measurements will—for the first time—relate the compatibility studies in the world of spectrum management to the emission limits in the world of electromagnetic compatibility. The impact that a lowered of increased EMC limit will have on the achieved utility of the spectrum would become visible and computable. This will help to take better informed decisions.

## Figures and Tables

**Table 1:** Number of outdoor measurements per country. The dominant contribution is highlighted in yellow.

Country	Nr of Meas.	Rel. nr. of Meas.
Germany	15,347	17%
Japan	71,796	80%
Netherlands	750	1%
Spain	22	0%
UK	2062	2%
USA	228	0%
total	90,205	

**Table 2:** Number of outdoor measurement locations per country. The dominant contribution is highlighted in yellow.

Country	Nr of Meas.	Rel. nr. of Meas.
Germany	654	79%
Japan	80	10%
The Netherlands	61	7%
Spain	1	0%
UK	33	4%
USA	4	0%
Total	833	

**Table 3:** Number of measurements per environment type.

Country	REMR	RURL	RESD	CITY	URBN	INDA	ROAD	OFFC	Other
**Germany**		1336	10,804	3207					
**Japan**			15,663	19,008	12,960	11,205	12,960		
**The Netherlands**	80	120	430	96					24
**Spain**	22								
**UK**		465	286	306	218	242	178	367	
**USA**				137	91				
**Total**	102	1921	27,183	22,754	13,269	11,447	13,138	367	24

**Table 4:** Relative number of measurements per environment type. The dominant contributions are highlighted in yellow.

Country	REMR	RURL	RESD	CITY	URBN	INDA	ROAD	OFFC
Germany		70%	40%	14%				
Japan			58%	84%	98%	98%	99%	
The Netherlands	78%	6%	2%	0.4%				
Spain	22%							
UK		24%	1%	1%	2%	2%	1%	100%
USA				0.6%	0.7%			

**Table 5:** Number of frequencies per country per environment type.

Country	REMR	RURL	RESD	CITY	URBN	INDA	ROAD	OFFC	Other
Germany		12	13	11					
Japan			14	12	12	12	12		
The Netherlands	28	12	12	12					12
Spain	1								
UK		2	2	2	2	2	2	2	
USA				3	2				

## Data Availability

Access to the data in the ITU Radio Noise Data Bank requires ITU membership.
